# The Human Brain Maintains Contradictory and Redundant Auditory Sensory Predictions

**DOI:** 10.1371/journal.pone.0053634

**Published:** 2013-01-07

**Authors:** Marika Pieszek, Andreas Widmann, Thomas Gruber, Erich Schröger

**Affiliations:** 1 Cognitive incl. Biological Psychology, Institute of Psychology, University of Leipzig, Leipzig, Germany; 2 Thomas Gruber, Experimental Psychology, Institute of Psychology, University of Osnabrück, Osnabrück, Germany; Baycrest Hospital, Canada

## Abstract

Computational and experimental research has revealed that auditory sensory predictions are derived from regularities of the current environment by using internal generative models. However, so far, what has not been addressed is how the auditory system handles situations giving rise to redundant or even contradictory predictions derived from different sources of information. To this end, we measured error signals in the event-related brain potentials (ERPs) in response to violations of auditory predictions. Sounds could be predicted on the basis of overall probability, i.e., one sound was presented frequently and another sound rarely. Furthermore, each sound was predicted by an informative visual cue. Participants’ task was to use the cue and to discriminate the two sounds as fast as possible. Violations of the probability based prediction (i.e., a rare sound) as well as violations of the visual-auditory prediction (i.e., an incongruent sound) elicited error signals in the ERPs (Mismatch Negativity [MMN] and Incongruency Response [IR]). Particular error signals were observed even in case the overall probability and the visual symbol predicted different sounds. That is, the auditory system concurrently maintains and tests contradictory predictions. Moreover, if the same sound was predicted, we observed an additive error signal (scalp potential and primary current density) equaling the sum of the specific error signals. Thus, the auditory system maintains and tolerates functionally independently represented redundant and contradictory predictions. We argue that the auditory system exploits all currently active regularities in order to optimally prepare for future events.

## Introduction

A conductor leading an orchestra relies on various information: Besides special knowledge about music theory and the musical piece being performed, the conductor listens to the music the instrumentalists are playing, watches musicians preparing for their contribution or playing their instrument, and concurrently follows the score including his/her annotations. One can derive predictions from these different sources of information about what is going to happen in order to act properly as well as to notice unexpected events. If something is not matching the prediction the conductor might initiate some adjustments.

In general, the generation of predictions from observable, learned relations in the sense of regularities of the environment seems to be one of the primary principles of the working brain [1]–[4]. In recent neuroscientific research it is commonly stated that the human brain operates in a predictive manner on different processing levels in order to improve adaptation to a dynamic environment. In every-day life, we encounter multimodal sensory information. For example, the sound of a drum cannot only be predicted on the basis of the preceding drum sounds but also by watching the movements of the drummer. The aim of the present study was to further characterize the functional architecture of the predictive brain. Specifically, we investigated the brain’s processing of auditory sensory predictions derived from auditory and visual information. We were interested in scenarios where auditory and visual regularities gave rise to the same auditory prediction and to divergent predictions. The presence of predictions was probed by measuring the brain’s error signals which are generated when auditory predictions are violated by the current sound.

Auditory predictions are even generated automatically (i.e., non-intentionally, on a pre-conscious processing level) on the basis of detected regularities of the acoustic world. One approach to study such automatically derived predictions utilizes the Mismatch Negativity component (MMN) of the event-related potential (ERP), [5]–[8]. The MMN is usually characterized as a negative, bilateral fronto-centrally distributed waveform in the range of 100–250 ms after stimulus onset. Its generators are located in the auditory and in the frontal cortex [9]–[11]. The MMN can be elicited, for example, in a so-called oddball paradigm by subtracting the ERPs of the frequently presented tones (the “standards” which confirm to the currently built regularity representations) from the rarely presented sounds (the “deviants” which do not match the representations of the standardś regularity). Assuming that each incoming sound is compared against a prediction derived from the present regularities [7], [12]–[14], the MMN can be interpreted as an error signal in the auditory system that is triggered whenever a sound violates a prediction.

However, auditory sensory predictions cannot only be established on the basis of auditory regularities but also on the basis of regular visual-auditory links [15]–[20]. For example, Widmann and colleagues (2004), [15], presented a simplified musical score in form of four to six visual bars. They occurred at two different vertical positions, corresponding to a low or a high frequency tone. The resulting melody was played to participants after short exposure to the visual “score”. ERPs elicited by tones being incongruent to the visual symbol (i.e., when the visual-auditory link was violated) revealed a negative going deflection relative to the ERPs to sounds being congruent to the visual score. This MMN-like so-called Incongruency Response (IR) was characterized by a fronto-lateral distribution in the latency range of 110–120 ms, indicating auditory sources. Thus, evidence from several studies suggests that sound prediction cannot only take place on the basis of auditory but also on visual information: Preceding and attended visual information that has previously been associated with auditory information mediates the processing of auditory input in a top-down manner.

Theories explaining the establishment of sensory auditory predictions often refer to the automatic, stimulus-driven extraction of regularities of the acoustic environment. Based on such regularity representations, internal generative models are derived which extrapolate future input, that is, predictions of forthcoming sounds from the encoded regularities [7], [20]–[22]. These theories further propose that the predictions are compared against the incoming sounds yielding a match or mismatch result. Provided the current auditory stimulus is matching the prediction, it requires only low processing effort as its processing is already prepared [4]. Also, it is part of the established generative model. However, a sound carrying novel information will not match the prediction. Therefore, the resulting prediction error signal (e.g., MMN) may indicate the emergence of a new regularity as well as weaken the confidence in the existing generative model. As a consequence, it modifies the model and further may trigger additional processing which may even result in an orienting response [4], [8], [10], [21]. This type of predictive coding theory formulated for regularities between auditory stimuli can easily be adopted for visual-auditory links [23], assuming that visual information is associated via implicit or explicit learning processes with auditory representations. On this basis, a preceding visual cue activates an auditory prediction. A forthcoming sound is then compared to this prediction.

Please note that this approach deliberately focuses on a situation in which the visual stimulation precedes the presentation of the sound to enable predictions to arise. We investigated how, where and particularly when visually processed information affects the processing of sounds. On the contrary, investigations with a simultaneous presentation of stimuli explore the when and where of multimodal integration and usually find interactive processes [18]–[19]. Several studies report integration starting already at the sensory level [24]–[27], whereas other studies show later interactions on the post-perceptual and pre-motor level [28]–[30], depending on materials, tasks, measured sites etc. The (posterior) Superior Temporal Sulci seem to be one of the most evident candidates for multimodal integration at a cortical level (e.g. [31]). We expected partly different networks to be involved and hypothesized not interactional, but additive (i.e. independent) predictive processing at the sensory level in the auditory system.

To learn more about the underlying mechanisms of auditory prediction processes, we examined scenarios in which concurrent independent auditory-auditory and visual-auditory regularities could give rise to opposing or to identical predictions. Ritter et al. (1999), [32], yielded evidence that the auditory sensory memory (as indexed by the MMN) and the higher-order attention-related processes (as mirrored in the P3) can generate opposite predictions. Using a cueing oddball paradigm, they demonstrated that the MMN is elicited even when the deviant is congruently cued by a visual stimulus and is, thus, consciously expected by the participants. Only at later processing stages the impact of prior visual information could be seen. We were interested in contradiction within the auditory system itself. We assumed that visual information could have an impact on auditory processing at this early stage (cf. [15]). This cannot be tested within the design by Ritter and colleagues [32] using only congruent cues. Hence, we modified this paradigm as follows: Sounds were presented with standard or deviant pitch. Additionally, the congruency of the preceding cue was manipulated (congruent vs. incongruent). This approach allows the dissociation of visually induced auditory predictions (visual-auditory, within-trial) and automatically generated auditory predictions indexed by the MMN (auditory-auditory, across-trials). Our design enables to probe the presence of contradictory predictions by occasionally violating either the auditory-auditory regularity (while obeying the visual-auditory regularity) or the visual-auditory regularity (while obeying the auditory-auditory regularity). More precise, this is the constellation of the visual source predicting the rare tone while the auditory-auditory regularity predicts the frequent tone (as it always does in oddball paradigms). We presented in one condition the rare tone vs. in another condition the frequent tone which confirms the one and violates the other prediction, depending on the tone. We expected the elicitation of a respective brain’s error signal in both conditions, that is, MMN in case the violated prediction was based on an auditory-auditory regularity and IR in case the violated prediction was based on a visual-auditory regularity. Moreover, the design allows the investigation of the processing of redundant predictions by occasionally violating both predictions, reflected in a component consisting of both IR and MMN. A redundant prediction emerges when the two independent predictive processes lead to the same result: two predictions with identical content (here always the frequent tone) are generated although one predictive process is sufficient to predict the sound.

More generally, we were interested in whether the independent auditory-auditory and visual-auditory regularities give rise to two independent generative models or whether only one is active. Previous research based on auditory-auditory regularities suggested that more than one regularity representation can be active. For example, by using regularities on different abstraction levels (global versus local rule) Horváth and colleagues (2001), [33], showed the simultaneous existence of regularity representations. Furthermore, for auditory streaming paradigms it is proposed that ambiguous sound organizations can compete and must therefore co-exist before one representation is selected [7]. The competition may be stopped to the benefit of the one generative model at the expense that the other generative model becomes inactive. To our knowledge, little is known about how the auditory system handles conflicting regularities. Also the reverse case, that is, when two independent regularities predicting the same sound are violated, has not yet been investigated systematically. From the principle that the auditory system tries to optimize processing effort [34], one may expect that when both regularities result in one and the same prediction, then only a single prediction is maintained and the redundant prediction is abolished. Further one might assume, the current sound is checked only once against this single prediction. However, from studies revealing that different features of a sound may be mismatched in parallel [35]–[36], indicating functionally and structurally independent feature representations, one may conclude that even the same sound can be checked twice against the same prediction(s) derived from different sources. Such a result could be expected if each of the two generative models checks the prediction independently from the other. Finally, our results could then support the assumption of - at least functional - independence of the predictions.

## Methods

### Ethics Statement

Participants gave their written informed consent according to the Declaration of Helsinki. Their data were analyzed anonymously. To separate participants from each other, ordinal numbers were assigned to them which did not include information about their identity. We followed the ethical guidelines of The German Psychological Society (“Deutsche Gesellschaft für Psychologie”, DGPs: http://www.dgps.de/dgps/aufgaben/ethikrl2004.pdf), thus, this experiment did not require any additional ethical approval.

### Participants

Eighteen healthy volunteers were paid or received course credits for their participation. All of them reported normal/corrected-to-normal vision and normal hearing. Two had to be excluded, firstly due to nonconformity with the instructions (participant reported afterwards to have not looked at the cues) and secondly due to poor task performance (participant did not sufficiently succeed to respond within the provided response time window). The data of the remaining sixteen participants (all right-handed; 8 men; mean age: 25.5 years, range 19–35) were included in the analysis. For the evaluation of the handedness we used a German short version of the Edinburgh Inventory [37].

### Materials and Procedure

**Figure 1:**
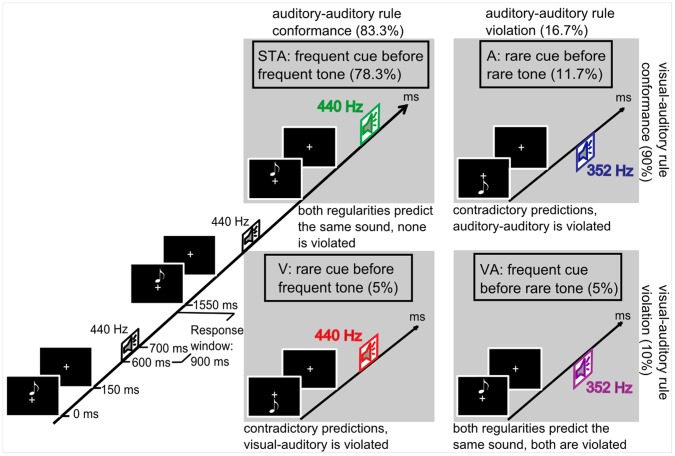
Illustration of the paradigm and systematic matrix of the four trial categories. Cue-sound combinations STA (frequent cue and tone, exemplarily displayed as high-pitched), A, V and VA are shown with their different probabilities. At least two standards precede each deviant as shown for type STA, the relevant tone is colored. The trial starts with the presentation of the high or low note symbol (cue), followed by one of the two tones (target) after an SOA of 600 ms. Type V and type A represent situations with contradictory auditorily and visually induced predictions whereas type VA means to violate redundant predictions from the two modalities.

The experiment was conducted in a sound-attenuated and electrically shielded chamber at the University of Leipzig. The stimulation was executed by Cogent Graphics toolbox (developed by John Romaya at the LON at the Wellcome Department of Imaging Neuroscience) via MatlabR2007b (The MathWorks., Inc.). A fixation cross, subtending a visual angle of 0.3°×0.3°, persisted during the stimulation slightly below the horizontal line of sight on a CRT screen 220 cm in front of the participant. Every trial started with the presentation of the visual stimulus for 150 ms. The cue was a white eighth note of 0.6°×0.9° visual angle presented with an eccentricity of 0.8° visual angle either above or below the fixation cross. The onset occurred 600 ms before the onset of the following auditory target ( = Stimulus Onset Asynchrony, SOA), see Figure 1. The two different triangle wave tones with base frequencies of 440 Hz and 352 Hz were presented binaurally via Sennheiser HD 25-1 headphones with an intensity of 70 dB SPL. The duration of each tone was 100 ms (including 10 ms rise and 10 ms fall times), the auditory stimulus onset asynchrony (ASOA) was 1550 ms. The frequency of the tones was balanced with the response buttons across participants, that is, participants were instructed to respond to the tone with the high pitch by pressing a key with the right thumb and to the tone with the low pitch with the left thumb, or vice versa. Additionally, the frequency of tones (high vs. low) defining the frequent (auditory standard) or the rare sound (auditory deviant) was counter-balanced (which therefore automatically applied to the cues). Thus, the number of participants has to be a multiple of four in order to fulfill the balancing. Participants were asked to attend to the cues and to use their informational content for a fast and correct response to the pitch. The response window started with the onset of the tone and lasted for 900 ms. Breaks were included on demand.

### Design

Target tones were presented according to an oddball paradigm with frequently occurring standard tones and infrequently occurring pitch deviants of 16.7% probability. The standard establishes by its high probability (83.3%) and isochronous presentation an auditory-auditory regularity. It could be either of high pitch for one half of the participants, or of low pitch for the other half (cf. balancing). One of two possible visual cues preceded each tone. The relation of the vertical position of the cue and the pitch of the tone lead to a visual-auditory regularity. In 90% of the trials the cue was in congruence with the target tone. Thus, predictions about the forthcoming tone could be based on the auditory-auditory regularity (which always predicts the frequent tone) and on the visual-auditory regularity (predicting the visually indicated pitch of the tone). This experimental protocol resulted in the following trial categories (see Figure 1): (1) STA: frequent cue before frequent tone (78.3%), obeying the visual-auditory and the auditory-auditory regularity and therefore resulting in a redundancy of auditory prediction; (2) deviant type A: rare cue before rare tone (11.7%). The visual-auditory regularity (prediction of the rare tone) is obeyed but the auditory-auditory regularity (prediction of the frequent tone) is violated by the presented rare tone. This is the one condition of contradictory predictions. (3) Deviant type V: rare cue before frequent tone (5%). The visual-auditory regularity (prediction of the rare tone) is violated but the auditory-auditory regularity (prediction of the frequent tone) is obeyed by the presented frequent tone. This reflects the other condition of contradictory predictions. Finally, (4) deviant type VA: frequent cue before rare tone (5%), that is, both visual-auditory regularity and auditory-auditory regularity predict likewise the frequent tone (i.e., redundant predictions are generated) but both are violated concurrently by the presented rare tone. The violation probabilities were chosen as they are to ensure that the participants keep the visual-auditory regularity also for the rare cues, as in two thirds of displaying the rare cue this link was confirmed (type A) but in one third a violation occurred (type V). 120 trials were presented in blocks of 3∶06 min duration. In total there were 18 blocks (2160 tones). The four trial categories were pseudo-randomized in each block; at least two standard trials (STA) were presented between two deviant trials (A or V or VA).

### Data Recording and Analysis

Reaction times and accuracy were measured, EEG was recorded with BioSemi amplifiers (BioSemi, Amsterdam, The Netherlands) at a digitization rate of 512 Hz. 32 electrodes were placed according to the international 10–20 system, and additionally at the tip of the nose, at the left (M1) and at the right mastoid (M2). The EOG was measured with one electrode at the nasion and two below the outer canthi of the eyes, according to the eye artifact correction procedure by [38]. The data were analyzed with the EEGLAB open source toolbox for Matlab [39]. The raw EEG was re-referenced to the nose and standards directly following a deviant trial were excluded from further analysis.

**Figure 2:**
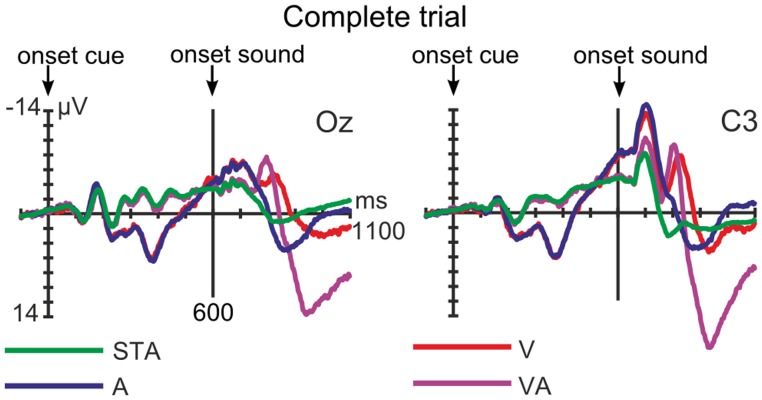
Grand-averaged, unfiltered ERPs of the complete trial (nose referenced). Cue and tone onset are marked for all four trial categories (green: frequent cue before frequent tone, STA; blue: rare cue before rare tone, A; red: rare cue before frequent tone, V; purple: frequent cue before rare tone, VA). Left: Visual ERPs to the cues are best observable in occipital electrodes (here Oz). Right: Please note that the data were not filtered to clearly show the CNVs which are influenced by the cue probabilities (more pronounced for the rare cue, type A and V). Tones were presented with an onset 600 ms after the trial started, eliciting auditory ERPs in fronto-central regions. Negative is plotted upwards.

For depicting the complete trial for all 4 categories (see Figure 2), the data epochs from −100 to 1100 ms relative to cue onset were averaged separately for each trial category. Afterwards, trials were rejected with amplitude changes exceeding 150 µV at any channel.

For the analysis of auditory electrophysiological data, the EEG was filtered offline with a 0.5–100 Hz bandpass FIR filter (1857 points, Kaiser windowed sinc FIR, Kaiser beta 5.65, [40]). Regression based EOG artifact correction was performed [38], afterwards the continuous data were filtered with a 1.3 Hz highpass filter (same properties as above). The EEG was averaged separately for each trial category and segmented into epochs, including a period of 100 ms previous to until 500 ms after the onset of the respective sound. Here also, epochs showing amplitude changes exceeding 150 µV at any channel were discarded. As depicted in Figure 2, right, the visual cues elicited Contingent Negative Variation potentials observable after the obligatory visual components until tone onset as a negative shift (see “Results” and “Discussion”). (A CNV is a slow anticipatory, frontally distributed negativity which arises if a target is regularly signaled by a cue and the participant is asked to respond to the target [41]–[42], demonstrating an expectation of and preparatory activity to the target.) They introduced a negative drift contaminating the pre-stimulus baseline of the auditory stimuli. CNVs could not be completely eliminated only by highpass filtering with a very high cutoff frequency as for example used by [29], [43]. Instead, we chose a cutoff frequency which avoided the distortion of the components indicating the violations of predictions. Such distortions are introduced by filtering the slow components (e.g., components of P3a and P3b can affect earlier and later components), [40], [42]. Having different latencies and amplitudes in the different conditions, slow components affect other components even differently due to filter artifacts. Additionally, as we did not expect measurable trial category related, non-oscillating effects within the first 50 ms following sound onset, we baseline-corrected the epochs relative to the 0 to +50 ms post sound onset interval.

For every deviance type, the associated deviant-minus-standard-difference waveforms were computed: A–STA, V–STA and VA–STA. These difference waves reveal the Mismatch Negativity (MMN), the Incongruency Response (IR), and the combined IRMMN, computed with the respective mean amplitude. Their peaks centered the corresponding time window of 105 and 130 ms, avoiding the contamination by the subsequent P2 component. Voltage distribution maps and scalp current densities (SCDs) were computed for the three deviant-minus-standard difference waves by using a spherical spline interpolation of the scalp potential data with a maximum degree of the Legendre polynomials of 50 and order of splines (m) of 4 [44]. A smoothing factor (lambda) of 10^–5^ was applied for the estimation of the SCDs. In order to localize the cortical generators of the MMN, IR and IRMMN, we applied VARETA (Variable Resolution Electromagnetic Tomography, [45]). This procedure provides the spatial intracranial distribution of primary current densities (PCD) in source space, which is best compatible with the amplitude distribution in electrode space. As possible sources of the signal 3244 grid points (“voxels”) of a 3D grid (7 mm grid spacing) were used. This grid and the arrangement of 32 electrodes according to the international 10–20 system were placed in registration with the average probabilistic MRI atlas (“average brain”) produced by the Montreal Neurological Institute (MNI; [46]). Statistical comparisons were carried out by means of Hotelling t^2^-Tests against zero. Activation threshold corrections, accounting for spatial dependencies between voxels, were calculated by means of Random Field Theory [47]. Regarding all statistical parametric maps (SPMs), the results were thresholded at a significance level of *p*<.01. Finally, the outcomes were depicted in the coronal slice containing the centre of gravity of the activation (constructed on the basis of the MNI average brain).

An additive model, a common procedure for difference effects in, for example, multimodal studies (A+V = AV; [28], [30], [48]), was applied to test our hypotheses. Therefore, the MMN and IR difference waves were added to a modelled IR+MMN wave. By comparing it with the measured IRMMN, the additivity of IR and MMN was checked. It should be noted that the criticism of Gondan and Röder (2006), [49], which applies to the additive model test of ERPs per se does not hold for difference waves. Crucially, a repeated-measures ANOVA was conducted. We included the individual mean amplitudes (105–130 ms) of the ERPs of standard (STA) and deviants A, V and VA of previously chosen regions of interests (ROIs lateral). ROIs were defined from electrodes with the maximum amplitudes in the difference potential by visual inspection to warrant the comparability between the effects. For the left hemisphere channels FC5 and C3 were included, for the right one channels FC6 and C4. To rule out significant differences in laterality, the factor of hemispheric distribution (2: left vs. right) was tested, together with auditory violation (2: auditory-auditory regularity vs. irregularity) and visual violation (2: visual-auditory regularity vs. irregularity) to test for interaction effects. Moreover, the additive model was tested for source strength by the addition of the PCDs of the centres of gravity of the VARETA, averaged for left and right hemisphere. Student´s *t*-test was applied to rule out significant differences between the modelled (IR+MMN) and the measured (IRMMN) response.

To test for interaction in the subsequent P2-range (131–175 ms), the repeated-measures ANOVA included the electrodes of Fz, Cz and Pz (ROI midline) with the factors auditory violation (2: auditory-auditory regularity vs. irregularity) and visual violation (2: visual-auditory regularity vs. irregularity). The ROI midline was also used for Student´s *t*-tests for the confirmation of significance from zero for components in the N2- (185–225 ms) and P3- range (235–355 ms) for the three deviant types. Additionally, the difference of N2 amplitudes for V vs. VA was tested at ROI midline with a paired *t*-test. The reaction times (RTs) and accuracy data were averaged for each trial category. Behavioral measures were tested with repeated-measures ANOVAs with the factors auditory violation (2: auditory-auditory regularity vs. irregularity) and visual violation (2: visual-auditory regularity vs. irregularity), followed up by paired *t*-tests (STA vs. A and V vs. VA). All reported results refer to a significance level of alpha = 0.05, two-tailed. To determine the relevance of the measured ANOVA effects for the electrophysiological data, both effect size measures of partial and generalized eta squared are included [50]. For reference of η_G_^2^ see Bakeman (2005), deriving 0.02 corresponds to a small, 0.13 to a medium and 0.26 to a large effect [51].

## Results

### The Complete Trial Including Visual ERPs, CNVs and Auditory ERPs

**Figure 3:**
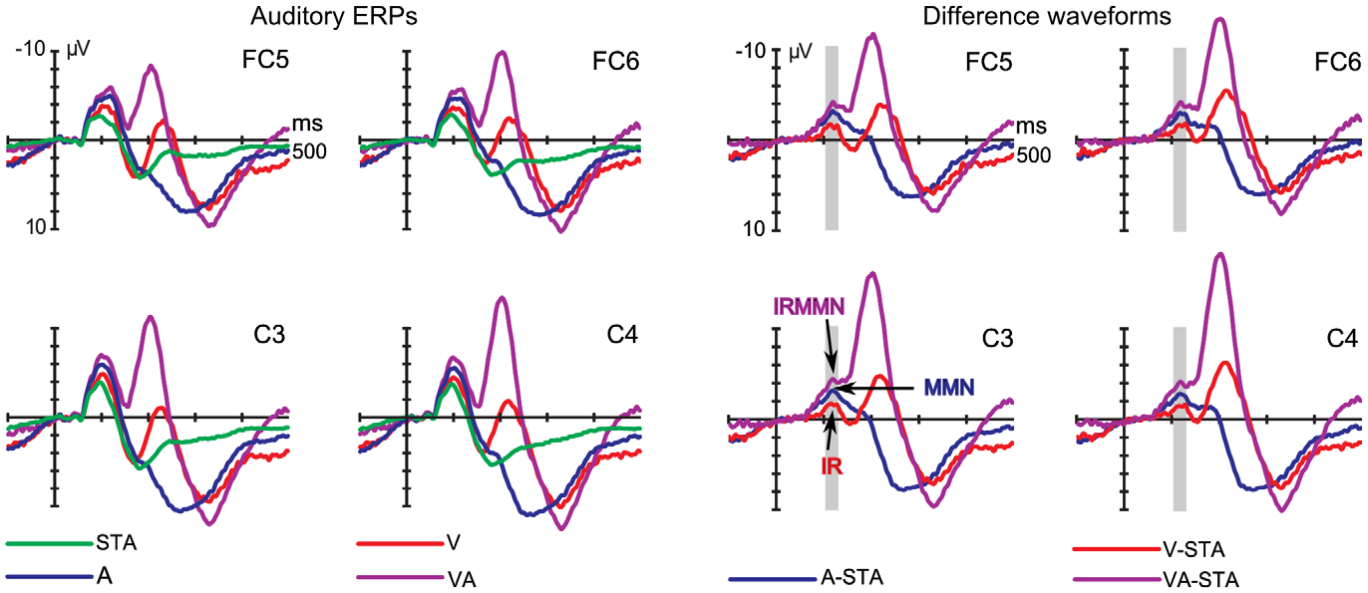
Filtered (1.3–100 Hz bandpass filter) and grand-averaged auditory ERPs and difference waves (nose referenced). Left: Auditory ERPs, elicited by the four types of cue-sound combinations (green: STA, meaning no violation; blue: A, meaning violation of auditory-auditory regularity; red: V, meaning violation of visual-auditory regularity; purple: VA, meaning concurrent violation of both regularities). Right: For every deviant the respective deviant-minus-standard difference waveform is shown (same filter setting and color code). The prediction error signals of MMN, IR and IRMMN correspond to the marked time window of 105–130 ms for the deviant types A, V and VA, respectively. Negative is plotted upwards.

Both cues elicited regular visual P1, N1 and P2 ERPs with the highest amplitudes in occipital areas, depicted in Figure 2, left. Two distinct Contingent Negative Variation (CNV) components could be seen in the following in central regions, Figure 2, right (cf. subheading “Data Recording and Analysis”). 600 ms after cue onset, a sound was presented which elicited for each trial category regular auditory ERPs (Figure 2, right). The corresponding 1.3–100 Hz filtered auditory ERPs as used in the analysis can be seen in Figure 3, left.

### The Auditory Difference Potentials Including Source Localization

In the time window of prediction error signals (105–130 ms) in ROIs lateral, the negative components of MMN, IR and IRMMN were reflected in the three corresponding deviant-minus-standard difference waveforms A-STA (violation of auditory-auditory regularity by rare tone), V-STA (violation of visual-auditory regularity by frequent tone) and VA-STA (violation of both regularities at the same time by rare tone), see Figure 3, right. Mean amplitude differences of the four trial categories were due to the two main effects of the factor visual violation (*F*(1,15) = 29.2, *p*<.001, *η_p_^2^ = *.67, *η_G_^2^ = *.08) and the factor auditory violation (*F*(1,15) = 55.8, *p*<.001, *η_p_^2^ = *.79, *η_G_^2^ = *.27). The interaction of both effects did not become statistically significant (auditory x visual violation; *F*(1,15) = 0.4, *p = *.551, *η_p_^2^ = *.02, *η_G_^2^ = *.003). We interpreted the small F-value and effect sizes as indicators of independence of the underlying prediction error processes, that is, additivity of the processes underlying IR and MMN.

**Figure 4:**
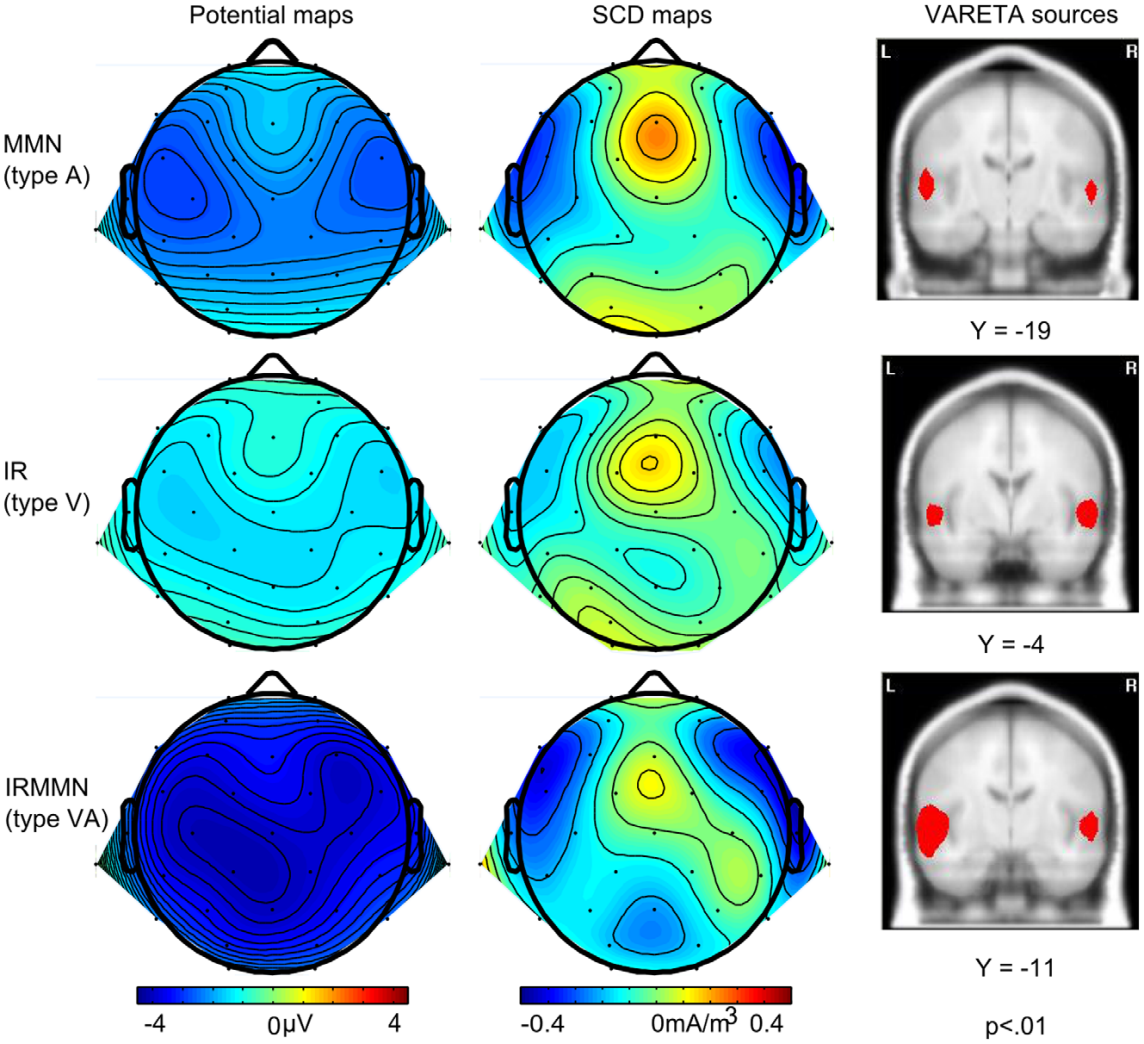
Topographic, SCD and VARETA SPM maps of the error signal components. Left: Potential maps (nose reference) show the scalp distributions of the difference data for MMN, IR and IRMMN (105–130 ms). Middle: SCDs for the three components of IR, MMN and IRMMN are compatible with sources in auditory areas of the temporal cortices. Right: SPMs of the source reconstructions of the three prediction error signals MMN, IR and IRMMN (*p*<.01). Coronal slices which contain the centre of gravity of the inverse solution are displayed (all within the Superior Temporal Gyrus). The Y-coordinates represent the location of the coronal slice in MNI space.

Effects of laterality could not be observed (factor hemispheric distribution; *F*(1,15) = 1.3, *p = *.277, *η_p_^2^ = *.08, *η_G_^2^ = *.005). The topographic maps (Figure 4, left) demonstrate similar bilateral voltage distributions of the three prediction error signals. The scalp current density maps (Figure 4, middle) show bilateral negative temporal sinks (and positive mastoidal sources for the IRMMN) which suggest auditory signal generation [10], [52]. Please note that the fronto-central source possibly corresponds to an activation of the attention-switching system (cf. [52], attend condition).

VARETA source analysis computed the mean PCD value of the centers of gravity, averaged for the left and right hemisphere in the time window of 105–130 ms. For MMN it was 1.05 µA/cm^2^ (standard error: 0.30), for IR 1.59 µA/cm^2^ (standard error: 0.40), and for IRMMN 2.44 µA/cm^2^ (standard error: 0.86). In Figure 4, right, the significant voxels are depicted for the three components of MMN, IR and IRMMN. VARETA source localization results support evidence for auditory generation of the error signals in the Superior Temporal Gyrus.

### The Additive Model

**Figure 5:**
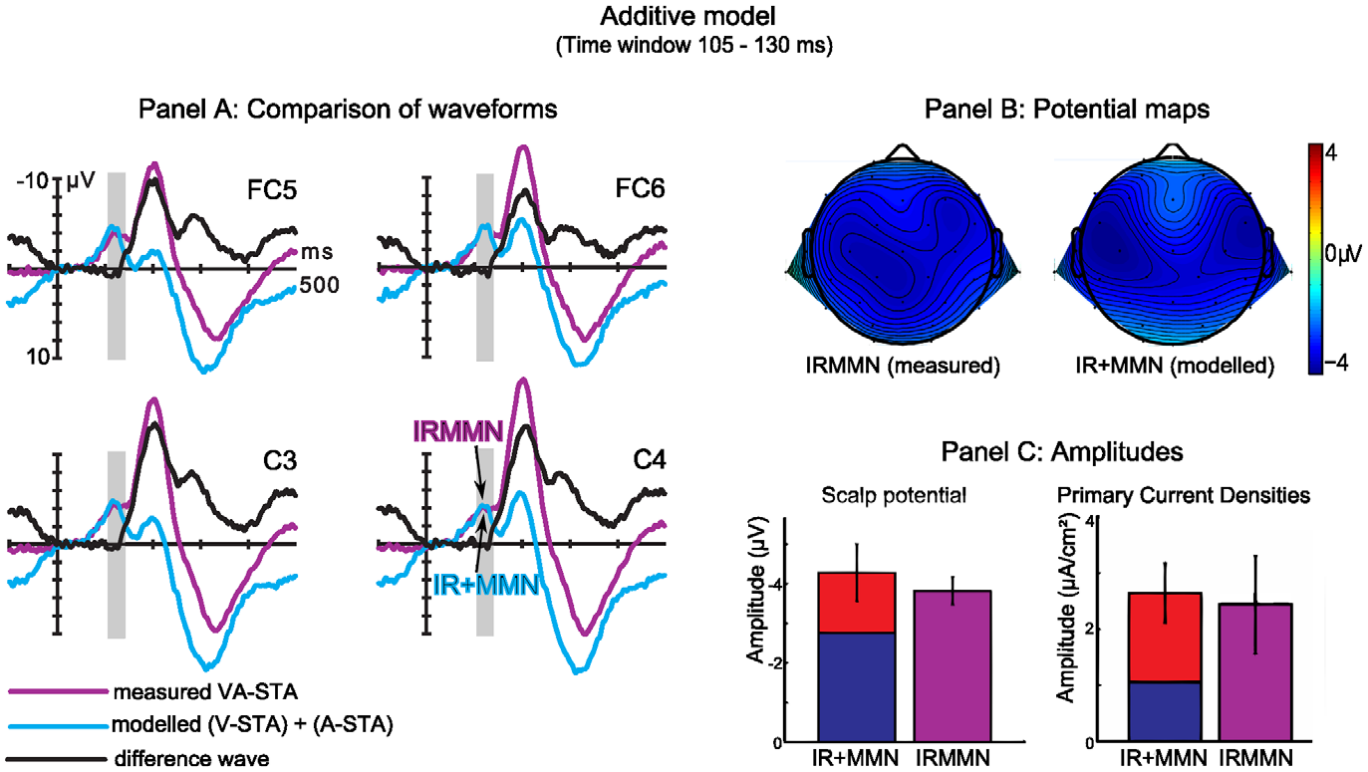
Illustration of the additive model. Panel A: The difference waveform (black line) of measured IRMMN (VA-STA) and the sum of the mean amplitudes of IR (V-STA) and MMN (A-STA) shows values around zero µV until the end of the time window of prediction error signals. Negative is plotted upwards. Together with the similar voltage distributions of measured and modelled component (panel B), this supports the assumption of functional independence of IR and MMN. Panel C: Bar graphs for potential and PCD data, showing amplitudes (left: mean amplitude of ROIs lateral) and PCDs (right) for modelled (IR+MMN) and measured (IRMMN) concurrent violation of sound prediction. For VARETA source analysis, mean PCD values of the centers of gravity were averaged for both hemispheres in the time window of 105–130 ms. Error bars represent the standard error of mean.

Figure 5 shows the application of the additive model. First, the results for the potential data can be viewed as following: In panel A, the difference waveforms of the concurrent violation of auditory predictions (measured IRMMN) are compared with the sum of the single violations (modelled IR+MMN). Their similar time courses and amplitudes (also depicted as bar graphs, see panel C, left) support the interpretation of linear additivity of the processes underlying IR and MMN elicitation. Spatially, their topographies seem also comparable (panel B).

Second, the source strength was computed by the PCD values of IR and MMN which add up to IR+MMN = 2.64 µA/cm^2^ (standard error: 0.53). Panel C, right, depicts that in comparison to the measured IRMNN (2.44 µA/cm^2^, standard error: 0.86) there is no difference (IR+MMN vs. IRMMN: *t*(15) = 0.33; *p = *.75), supporting the assumption of additivity as well.

### The Subsequent Processing of Tones

Already at P2 latency (131–175 ms), significant interactions were found (auditory vs. visual violation; *F*(1,15) = 15.9, *p = *.001), therefore an additivity was not given any longer. Subsequently, in the N2 range, a negative fronto-central, slightly right-hemispheric component was elicited only for the tones which violated the visual-auditory regularity (type V: *t*(15) = −5.6, *p*<.001; type VA: *t*(15) = −9.1, *p*<.001) with a significant difference in amplitudes between tones of V and VA trials (*t*(15) = 6.9, *p*<.001). P3 components (235–355 ms) were obtained for all three deviant types (type A: *t*(15) = 4.0, *p = *.001; type V: *t*(15) = 5.6, *p*<.001; type VA: *t*(15) = 8.4, *p*<.001). Whereas P3 components to deviants V and VA had a frontal distribution, P3 to the rare tone violating the auditory-auditory regularity (type A) was distributed parietally (cf. Figure S1).

### The Behavioral Measures

**Figure 6:**
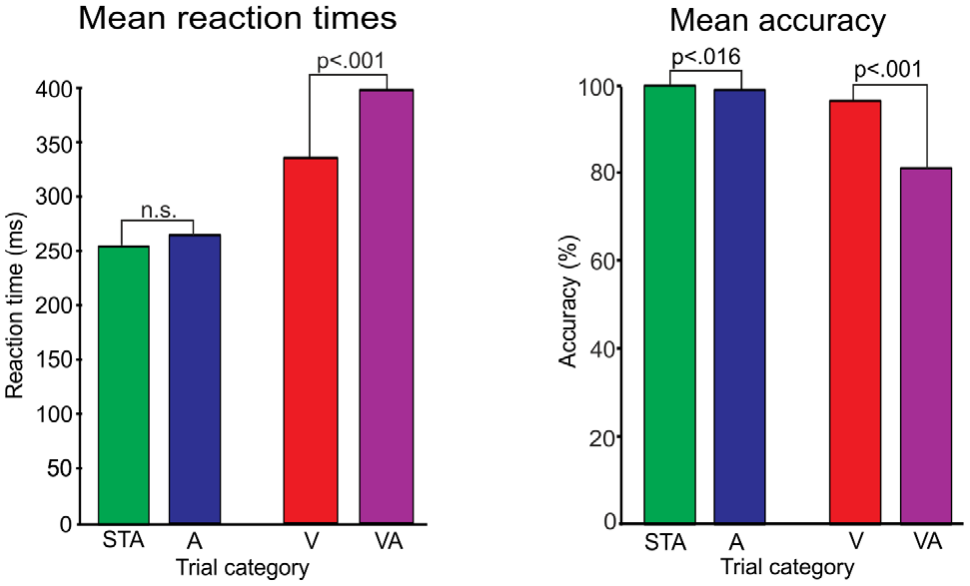
RTs (left) and accuracy data (right). Tested pairs with their significance levels are tagged (STA: frequent cue before frequent tone vs. A: rare cue before rare tone; V: rare cue before frequent tone vs. VA: frequent cue before rare tone).

98.16% of responses were given within the defined time window and included in further analyses. Reaction times and accuracy data are shown in Figure 6. For RTs, a significant interaction (auditory x visual violation; *F*(1,15) = 32.8, *p*<.001) as well as main effects for factor auditory violation (*F*(1,15) = 38.5, *p*<.001) and factor visual violation (*F*(1,15) = 455.1, *p*<.001) were obtained. Auditory violation alone had no significant impact on RTs (*t*(15) = −1.3, *p = *.22) but a significant impact when paired with a visual violation (*t*(15) = −8.5, *p*<.001). That is, auditory and visual violation had a super-additive effect when combined. The analysis of accuracy also yielded a significant interaction of both factors (auditory x visual violation; *F(*1,15) = 30.8, *p*<.001) as well as main effects for the factors auditory (*F*(1,15) = 27.4, *p*<.001) and visual violation (*F*(1,15) = 31.7, *p*<.001). The significant differences between both comparisons of categories STA vs. A (*t*(15) = 2.7, *p = *.016) and V vs. VA (*t*(15) = 5.4, *p*<.001) showed a mutual influence of factors auditory and visual violation.

## Discussion

We determined how the auditory system handles the violations of redundant and of contradictory auditory predictions derived from independent auditory-auditory and visual-auditory regularities. In our cueing oddball paradigm, high- and low-pitched tones were presented, the one serving as auditory standard, the other as auditory deviant. Each tone was preceded by a note symbol being indicative with respect to the pitch of the forthcoming tone in the majority of the trials. There were trials in which the automatic generative model based on the auditory-auditory regularity and the generative model based on the attended visual-auditory regularity predicted the same sound (e.g., a low-pitch tone), and there were trials in which they predicted different sounds (i.e., the visual-auditory regularity predicted a high-pitch tone whereas the auditory-auditory regularity predicted a low-pitch tone). In the following, we will shortly discuss the error signals obtained when a prediction was violated. The evaluation of the results is separated for conflicting and redundant predictions situations with respect to what they tell us about the characteristics of generative models based on independent regularities. Subsequently, the later processing stages are shortly described to capture the further steps in processing.

### Signs of Prediction and Prediction Errors

First, the presence of the CNVs demonstrates the preparation to the predicted tone based on the visual-auditory link. Their distinct expression to the rare and the frequent cues might be related to a response preference for the frequent cue. Hence, CNV is an indicator of expectation of an event to occur but it is confounded with task-related processes [41]–[42]. Moreover, in this latency range we cannot infer anything about the across-trial predictions derived only on the basis of the auditory input.

Thus, focusing on the more informative processes after tone onset, we find that each of the three types of prediction violation elicited an error signal being visible in the deviant-minus-standard difference waveforms at a latency range of 105 to 130 ms: The MMN indicating the prediction error for rare tones breaking the auditory-auditory regularity only (A), the IR indicating the prediction error for tones violating the visual-auditory regularity only (V) and the IRMMN being elicited by sounds violating the prediction generated from both regularities (VA).

Before interpreting the results in more detail, a characterization of the components seems useful. Beginning with the MMN, its topography seems roughly comparable to usual frequency MMN topographies in active, only-auditory paradigms (cf. [52], attend condition, 220 ms). Its SCD map showed comparable bilateral fronto-temporal sinks and posterior-temporal sources to only-auditory studies [52]–[53], as do VARETA sources. They were located bilaterally in the Superior Temporal Gyrus, a typical finding for auditory MMN elicitation [54]–[56].

Crucially, the latency and distribution of the IR mirrored the one obtained in the previous IR study by [15]. Additionally, the present study found VARETA sources in auditory areas showing that the IR most likely is an auditory response. This conclusion is supported by its corresponding SCD map which is comparable to the SCD map of the present MMN. This supports the model provided by Widmann and colleagues (2007), [23], postulating that the visual symbols activate auditory memory representations of the to-be-expected sound. Hence, we can assume predictive mechanisms in the auditory system. We cannot exclude audiovisual integration processes despite the visual-auditory input delay which can take place already in the Superior Colliculi [57].

The amplitude, the time course and roughly the spatial distribution of the scalp potential of the IRMMN equaled the sum of the IR and MMN. The source activation strength of the VARETA solutions also argues in favor of the additive model, meaning that the IRMMN is composed of simultaneously elicited IR and MMN. In other words, the brain processes violations of the auditory predictions based on the auditory-auditory and on the visual-auditory regularities separately. Thus, the processes underlying MMN do not affect the co-occurring processes underlying IR and vice versa. Importantly, it might be considered that the observed independent representations of auditory and visual predictions must not necessarily be realized in different subsystems or neural substrates. MMN elicitation can also be explained by modulation of the ERP response to standards consisting of passive (e.g., refractoriness) as well as active processes (e.g., lateral inhibition). Both processes pre-activate neurons generating the N1 component which result in N1 suppression and delay [58]–[59]. A model assuming that (a) these neurons can also be pre-activated in a top-down manner by visual predictive information and (b) that pre-activations can be additive would be fully compatible with the observed data pattern but realized in a single system. Therefore, we propose a *functional* independence or co-existence of the underlying mechanisms. To draw conclusions regarding an anatomical independence an imaging study with higher spatial resolution would be required. The functional relevance of this finding for our understanding of the auditory generative models will be discussed under the subheading “Redundant Predictions”.

Alternatively, the results can also be explained in terms of the interaction between the overall probability of the sounds and the conditional probabilities, i.e. separately focusing on the frequent or the rare cue and their related tone probabilities. The resulting probabilities of visual-auditory events only might modulate the N1 differently, reflected in the order of the amplitude sizes of the four conditions (see Figure 3, left). However, this alternative interpretation lacks the functional aspect we aim at. The functional interpretation as given above is fully compatible with the theory of predictive coding [60]–[62], suggesting a pre-activation of neurons by a prediction via backward connections. This leads to suppression of their activity and therefore the suppression of the prediction error. If a stimulus carries information deviating from the backward prediction (here: top-down), a forward error signal is produced activating neurons at other hierarchical levels. The possible existence of “prediction error units” [62], as assumed for e.g. eliciting MMN, explains convincingly the occurrence of a bigger error signal if there are two violations compared to one as in the present study.

### Conflicting Predictions

In A and in V trials, the generative models based on the auditory-auditory regularity and the visual-auditory regularity result in different predictions: For example, the one predicts a high-pitch sound, while the other predicts a low-pitch sound. Given that only high- and low-pitch tones were presented, that the participants were instructed and that they had learned that only one out of two different sounds can be presented at a time, these predictions can be called contradictory. In other words, the prediction for a high-pitch tone implies that the next sound is not expected to be a low-pitch tone, and vice versa. However, as MMN was obtained in A trials *and* IR in V trials, the system had obviously concurrently maintained both contradictory predictions at this processing stage.

This conclusion implies firstly the existence of multiple predictions which is in line with current research. Evidence from empirical [7], [33] and computational modelling studies (Mill RW, Böhm TM, Bendixen A, Winkler I, Denham SL (unpublished) Modelling the emergence and dynamics of perceptual organization in auditory streaming) suggest that more than one regularity representation can be active. An ongoing competition is supposed to last until one of the alternative representations is selected, presumably at a stage before MMN is elicited [7]. If the point in time was valid for the present study, an error signal should have been elicited only in A vs. only in V trials but not in both conditions as observed. For instance, the auditory-auditory predictive model might overrule the other because of a general preference of the inherent sort of input (i.e., auditory in the auditory system) or because the auditory system might be an encapsulated structure (i.e., stimulus-driven, modular, cf. [32]). Here, only the MMN should be elicited by the sound of deviant type A but no IR by type V. The other way round, a visually aligned perception, based on quite reliable, attended prior information, might have dominated the processing already here (as conceptualized for the later N2 level, see subheading “The Impact on Subsequent Processing Stages and the Outcome”). This would have led to an IR in V trials but to no MMN in A trials. In our paradigm, there are two predictions maintained concurrently still at the “MMN stage”, selection does not take place here.

Secondly, our conclusion implies that auditory processing can be influenced already at this processing stage from top-down information despite the findings of some auditory studies utilizing MMN as a well-known indicator for early cortical auditory-sensory processes. For example, the MMN was found not to be modulated when the listener was informed about a forthcoming deviant [32], [63]–[64], whereas the subsequent P3 stage was affected by the predictive information (via a visual cue), [32], [64]. Thus in deviant trials, the lower order cognitive system underlying the elicitation of the MMN predicted an auditory standard, while the higher order cognitive system underlying the elicitation of the P3 predicted an auditory deviant, using the information from other than auditory sources. Hence, these findings were interpreted in terms of an encapsulation of the processes underlying MMN, further the authors concluded that the brain can generate opposing predictions. Critically, there was no independent manipulation of the congruency of the cue but only its specificity was varied in a predictable vs. unpredictable condition. Our study, in turn, cannot focus on different manifestations in MMN- and P3-“systems” as it is lacking an unpredictable condition, but it reveals that the presence of conflicting predictions is not confined to processing systems organized at different cognitive levels. The MMN and the IR were overlapping in time and both were generated in auditory areas. This relates to where the error signal has been generated and likely to where the respective predictions are represented.

To sum up, we could show that auditory processing at this stage is affected by prior knowledge, that is, by top-down information from other sources. We can integrate these systematic results with previous findings by showing again that the auditory MMN is elicited in a modular manner but that there is another, functionally independent prediction for sounds derived via non-auditory information. As a side note, one should consider that the visual-auditory regularities were made explicit via instruction which may have induced respective bindings across modalities [15]. As feature overlaps can be encoded across modalities even when they are not task relevant [65], it seems possible that such regularities can also be acquired incidentally and may then result in automatically generated predictions. However, as long as the automatic (“pre-attentive”) elicitation of the IR is not shown, we prefer to separate IR and MMN for the moment. Both components are elicited in completely different contexts, i.e. paradigms (oddball vs. symbol-to-sound matching) and attentional conditions. Nevertheless, it seems likely that an IR reflects the same brain processes and structures also related to the MMN component but measured in a different context.

### Identical Predictions Leading to Redundant Information

In STA and VA trials, both the auditory-auditory and the visual-auditory regularity predicted the same sound. In other words, both generative models put forward identical predictions. In VA trials, both were violated at the same time, leading to the elicitation of the IRMMN. The additivity analyses of the ERP difference waves and the PCDs suggested that the IRMMN is, in fact, composed of concurrently elicited error signals. That is, in VA trials the same MMN is elicited than in A trials and the same IR is elicited than in V trials. This suggests that the current input is compared to both generative models in parallel. We cannot infer from our data whether there exist two independent representations, one from each generative model, which represent the same sound and which are mismatched in parallel, or whether there exists only a single cumulatively operating representation to which each generative model has independent access for the comparison.

The finding of the present study can be related to auditory MMN studies showing that the MMN for different sound features such as frequency and location are elicited independently from each other (the respective representations or, at least, the error signals could be shown or inferred as even located in slightly different areas of the brain), [35]–[36], [66]–[68]. While this makes perfect sense with respect to different features of a sound, it seems, at a first glance, rather surprising in our experimental scenario. However, from the auditory streaming literature [69] it is known that several alternative regularity representations describing a sound sequence can co-exist [7].

### The Impact on Subsequent Processing Stages and the Outcome

It should be noted that additivity was only present in the time range of MMN and IR. At later processing stages (P2, N2, P3) there were distinct interactions. N2 elicited in VA trials was by far larger than the sum of the N2s obtained in A and V trials. N2 was also visible in V trials but there was no (or only a very small one) in A trials. Usually, the N2 elicitation in oddball paradigms is assumed to reflect attentive deviant detection [70]–[72]. Hence, we had expected the participants to consider auditory deviants in A trials as targets due to their task (requiring a different response from the frequent tone and response). Tentatively, the absence of N2 in A trials suggests that the visual-auditory model was assessed as rather reliable and might therefore have suppressed the lower-level, automatically gained auditory-auditory regularity representation. On this higher, presumably conscious level the processing was already prevailed by the attended, prior visual information (not as late as at P3-level as reported by [32], [64]). Hence, the auditory deviant was not or less “surprising” as it was indicated before by the cue; in the following, the already pre-activated response (by the cue) can be prepared correctly.

For incongruent trials (V and VA), the cue led to a wrong sound prediction (and response preparatory processes). Participants have attentively detected the incongruence between cue and tone in most cases as mirrored by the accuracy data. Thus, these salient events may have been regarded as the deviants and the elicitation of N2 might be explained as the result of conscious deviance detection, independently of the respective distinct response which was required in V and VA trials. Remarkably, the N2 in VA trials was about three times as large as in V trials. In VA trials, both predictive models predicted likewise the frequent sound; therefore, the presence of an additional violation (of the auditory-auditory regularity) to the visual-auditory violation presumably fostered the processes underlying the N2, compared to V trials with an only visual-auditory violation. Thus, for VA types none of the models is suppressed, auditory-auditory information still seems to affect conscious processing when being redundant. From our data we cannot infer at what point in the time course the response preparation gets disturbed. Studies investigating processes of cognitive control with go/no-go tasks report also N2 elicitation/modulation when participants inhibit a required reaction to a cue, that is, when a response conflict is detected and participants adapt their behavior [73]–[74]. The paradigm used in the present study differs from those studies, but nevertheless we assume also processes of cognitive control to enter about here which might also modulate N2b [cf. 75–76].

The performance loss resulting from a disturbed preparation of action is best observable for RTs and accuracy in VA trials. The finding of the slowest RTs for the pitch discrimination task could be interpreted in the context of the redundant-targets effect [77], which consists in decreased reaction times to audiovisual redundant targets. Here, the present double violation of the identical predictions resulted in an expected opposite effect. The mutually influenced slowing of the response times occurred due to an impaired response preparation according to both predictive models.

Moreover, one could reframe the N2 and behavioral results by relating them to the different cue probabilities. Internally, the frequent stimuli may result in labelling the required responses as “non-target responses” linked to the frequently used hand, while rare-stimuli responses might be labeled as “target responses” linked to the rarely used hand. Thus, an internal preference may be established for the frequent response. Therefore, the highest N2 amplitudes, slowest RT and lowest accuracy in VA trials can be explained by the fact that the rare sound (“target”) was cued as a non-target (“frequent”). High performance costs result from two reasons: The formed expectation by the actual cue and the preferred, all-time activated “frequent” response are not only both wrong and useless at once without any other weakening or suppressing influences, but a new response has to be prepared. On the contrary in V trials, the lower amplitude for N2, coupled with intermediate RT and accuracy, may be due to a partly suppression of the preferred response by the (misleading) rare cue. Here, a target response (“rare”) is expected. Further, the processing and the response preparation may be more efficient in trials with congruent links (STA and A) due to the quite reliable cues. RTs are lowest and do not differ. Especially the usual costs in an attended oddball paradigm, i.e. the slowing of RTs for the deviants, and N2 elicitation are diminished by the predictability due to the visual cue. Still it remains unclear, why the overall probability and the response selection for the target (“rare”) affect RTs and N2 only marginally (cf. “Results”: auditory violation alone had no impact on the RTs, but on accuracy). In these terms, this explanation seems not sufficient: In A trials, the rare cue weakens the preferred response (like in V trials). Additionally, the actual rare sound (like in VA) makes the preferred response even obsolete which should be reflected in the data by a higher N2 response and behavioral performance loss. Suddenly, the influence of the cue seems to be very strong compared to the other conditions in gating the processing.

Rather, the incongruent cue might lead to a disturbed encoding and failed recognition of the upcoming auditory event (cf. [78]: creation of a stimulus set by a cue-target association). The system was set to process the other tone, respectively, like indicated by different CNVs for frequent and rare cues. In addition, the audiovisual incongruency may prevent a correct preparation for an intentional action: Gratton et al. (1990), [78], found that the cue establishes a response set (creation of a cue-response association) independently of the stimulus set. Subjects prepare for the indicated required response without being biased by the “frequent” response, especially when a cue predicts one of the two events more likely than the other event [78], as realized in the present study.

Finally, all deviant types elicited a P3. P3 components indicate the processing of prediction violations at a later stage, possibly related to involuntary attention switch [10] or mobilization for action [79], or the evaluation of a sound that has been flagged as violating a prediction (Winkler I, Schröger E (in revision) Predictive regularity representations in service of object formation and new information detection in audition).

## Conclusions

The presence of error signals shows that auditory predictions can be derived from different kinds of detected regularities of the current multimodal environment. Auditory predictions are not only derived from existing regularities within the auditory world. They can also be made on the basis of visual-auditory links, even in environments which contain both kinds of regularities. Moreover, the results obtained for redundant predictions and for contradictory predictions suggest that the respective internal generative models based on auditory-auditory regularities and on visual-auditory regularities operate in a highly functionally modular fashion. Interactions indexing common processes are located at later processing levels, reflected by P2 and N2 components.

On the one hand, inconsistencies between the resulting predictions are not dissolved at the level of the generative models as reflected in the IR and MMN error signals. Thus, the information processing is not prevailed by a selected model but rather by the co-existence of models. On the other hand, when two different generative models predict the same sound, there seems to be a double comparison for the prediction as the same two error signals are elicited that occur when only the auditory-auditory or only the visual-auditory regularity is violated, reflected by the additive model. One may argue that the lack of a consistency check between incompatible predictions and the presence of a double check for redundant predictions is not a very parsimonious way to model regularities and to deal with violations of derived predictions. However, we interpret these characteristics of the generative models as an advantage with respect to goal-directed behavior. Such a system is able to exploit all information available in order to be prepared for the possible, because probable, future events. Of course, it is very likely that alternative generative models are compared against each other and that unsuccessful models become obsolete sooner or later ([7]; in the present study, latest at N2 level the likely dominance of the prior, attended, rather reliable visual information can be observed). However, in our experimental scenario both regularities were valid and the system behaved in a rational manner.

## Supporting Information

Figure S1 **Topographies of the P3 components of the differential potentials of A-STA (left), V-STA (middle) and VA-STA (right) in the time window of 235–355 ms.**
(TIF)
