# Correction: The Human Brain Maintains Contradictory and Redundant Auditory Sensory Predictions

**DOI:** 10.1371/annotation/c7efeea0-cd8e-4c37-ae70-1a97702833a2

**Published:** 2013-09-09

**Authors:** Marika Pieszek, Andreas Widmann, Thomas Gruber, Erich Schröger

A webpage describing the project supported by the Deutsche Forschungsgemeinschaft grant can be found with the following link: http://www.uni-leipzig.de/~biocog/content/en/third-party-projects/koselleck.

