# Supplementary figures and images for: The Human Brain Maintains Contradictory and Redundant Auditory Sensory Predictions
Source: PLoS One. 2013 Jan 7;8(1):e53634. doi: 10.1371/journal.pone.0053634 (PMC3538730; doi:10.1371/journal.pone.0053634)

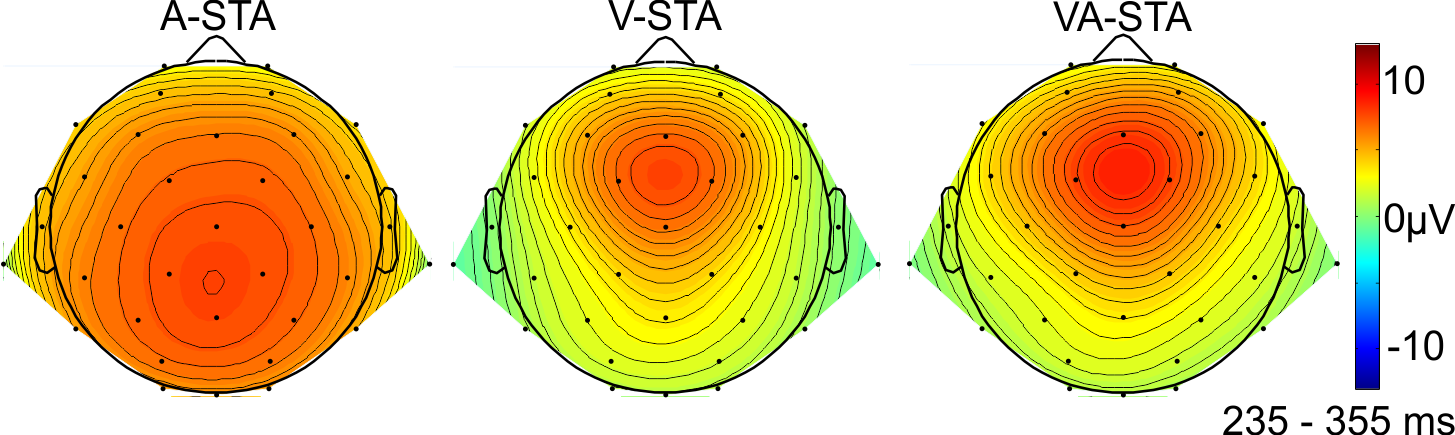

Supplement: Figure S1 — Topographies of the P3 components of the differential potentials of A-STA (left), V-STA (middle) and VA-STA (right) in the time window of 235–355 ms. (TIF) [file pone.0053634.s001.tif]
